# Molecular virulence determinants of *Magnaporthe oryzae*: disease pathogenesis and recent interventions for disease management in rice plant

**DOI:** 10.1080/21501203.2020.1868594

**Published:** 2021-01-15

**Authors:** Lovely Gupta, Maansi Vermani, Simran Kaur Ahluwalia, Pooja Vijayaraghavan

**Affiliations:** Anti-mycotic and Drug Susceptibility Lab, Amity Institute of Biotechnology, Amity University, Noida, India

**Keywords:** *Magnaporthe oryzae*, rice blast, virulence genes, pesticides, mutant breeding

## Abstract

*Magnaporthe oryzae*, causative agent of the rice blast disease, is a major concern for the loss in yield of rice crop across the globe. It is known for its characteristic melanised dome-shaped appressorium containing a dense melanin layer. The melanised layer is of considerable importance as it is required to generate turgor pressure for initiating peg formation, consequently rupturing the plant cuticle. Various virulence factors play an important role in the disease progression as well as pathogenesis of the fungus. Some of the proteins encoded by virulence genes are associated with signalling, secondary metabolism, protein deprivation, defence responses and conidiation. The purpose of this review is to describe various fungal virulence determinants and provide insights into the molecular mechanisms that are involved in progression of the disease. Besides, the recent molecular approaches being employed to combat the rice blast have also been elaborated.

## Introduction

Rice (*Oryza sativa*) is an essential food crop consumed by over 3.5 billion people across the world (Godfray et al. 2010). It is also an economically important crop as it is employed in textile, leather, cosmetics and food industry. However, pathogens such as fungi, bacteria, viruses and nematodes infect rice plants, causing a substantial loss in its yield (Asibi et al. 2019). Rice blast is one of the most significant and devastating disease occurring in the rice crop leading to almost 10% to 30% of crop loss every year (Sakulkoo et al. 2018).

**Figure 1.**
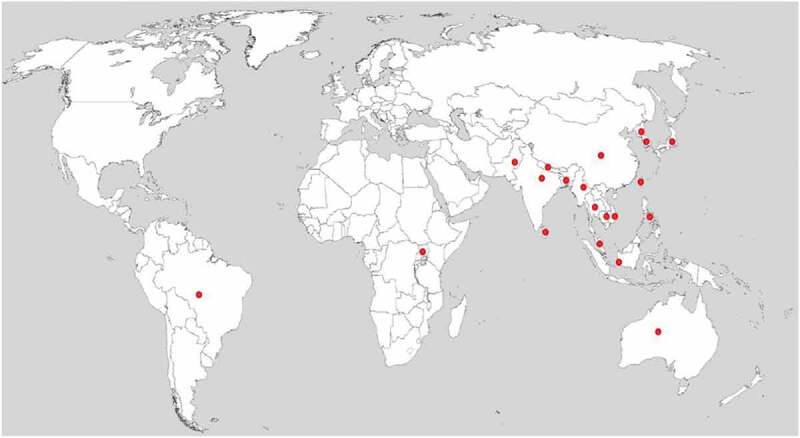
Global distribution of rice blast disease. Red spots indicate the countries with reports of rice blast

Rice blast disease is caused by fungi *Magnaporthe oryzae*. It affects the foliar parts of the plant at the sprouting and mature stages. Rice cultivated in temperate and subtropical climates of Asia are highly vulnerable to this pathogen, while rice cultivation in tropical upland areas is prone to this pathogen only under irrigation (Nutsugah et al. 2008). The disease spreads frequently under moist conditions with relative humidity ranging from 80%-100% and temperatures ranging from 25 to 30°C (Talbot 2003). Significant harvest losses have been reported in many rice-growing countries of the South-east Asia (China, Sri Lanka, Indonesia, Bangladesh, India) (Wilson and Talbot 2009; Suprapta and Khalimi 2012; Kumar and Kalita 2017) and also in other regions like South America, Australia, Korea and Philippines (Greer and Webster 2001; Pena et al. 2007; Shahriar et al. 2020) (Figure 1). In an outbreak of rice blast disease in Malaysia, yield loss caused by panicle blast was as high as 50–70% (Ashkani et al. 2015; Zakaria and Misman 2018). Zhang et al. (2010) reported 40–50% rice yield loss due to blast infection in China. Yashaswini et al. (2017) reported blast disease percentage ranged from 50 to 74% in different districts of Telangana and Andhra Pradesh in India. Similar study has been reported from eastern India where rainfed rice yield loss was around 30% per annum (Jha et al. 2012).

## Pathogenesis and infection cycle of *Magnaporthe oryzae*

Rice blast symptoms rely on ecological conditions age and the level of resistance of the host plant. The pathogen predominantly infects foliage, causing blasting during vegetative growth phase, or in reproductive stage on the necks and panicles (Shahriar et al. 2020). These indications are extreme in case of blast of neck usually defined by the infection at panicle’s base and its rotting.

**Figure 2.**
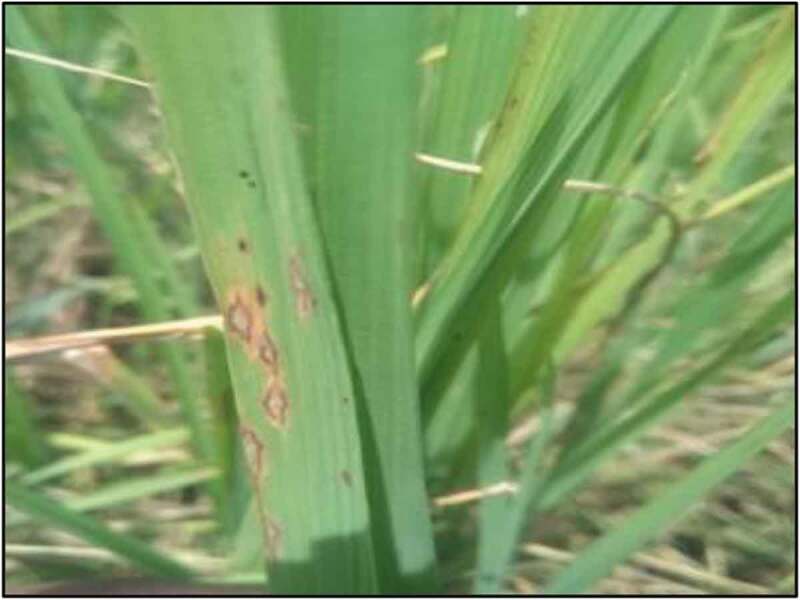
Rice blast lesions on a leaf of rice plant

The disease is caused by a heterothallic ascomycete *M. oryzae* that produces asexual or sexual spores (ascospores) in structures called asci (Wilson and Talbot 2009). The pathogen initiates infection through a protracted biotrophic stage, where the fungus bounded by the invaginated plant plasma membrane, grows within host cells and proceeds to a necrotrophic stage leading to lesion development (Figure 2). The mycelium consists of branched, septate and uninucleate hyphae. Septate conidiophores with a dark base forming hyaline and pyriform conidia acrogenously. The pathogen produces lesions on leaves (leaf blast), leaf collars (collar blast), culms, culm nodes, panicle neck nodes (neck rot), and panicles (panicle blast). These lesions vary in colour and shape depending on environmental conditions and developmental stage of the plant (Law et al. 2017).

**Figure 3.**
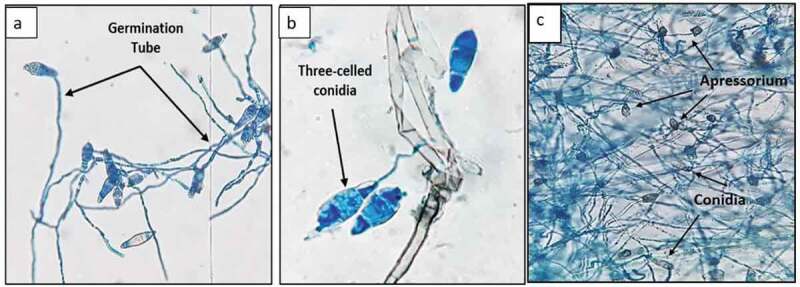
Microscopic view of *M. oryzae* (a) and (b) three-celled conidia with germination tube (40× magnification); and (c) appressorium formation (10× magnification)

The infection begins when a three-celled conidium (Figures 3 (a,b)) comes in contact with the hydrophobic surface of the leaf and attaches itself by an adhesive called spore tip mucilage (Wilson and Talbot 2009). The germ tube formed after germination of conidia swells into appressorium (Figure 3 (c)), a specialised infection structure capable of penetrating the leaves and stems of the rice plant. As the appressorium undergoes maturation, a dense layer of melanin accumulates in the appressorium wall (Boddy 2016). Subsequently, hydrostatic turgor pressure of up to 8 MPa develops due to accumulation of glycerol in the appressorium, which provides sufficient mechanical force to perforate the leaf cuticle and enter the plant epidermal cells with the help of penetration peg (arising at the base of appressorium). Eventually, invasive hyphae prevent nutrients and water from reaching the kernels and also secrete effector molecules to suppress host immunity and aid infection (Giraldo et al. 2013). These effector molecules move into host cell cytoplasm by a biotrophic interfacial complex, a plant-derived membrane-rich structure (Giraldo et al. 2013; Mochizuki et al. 2015).

**Figure 4.**
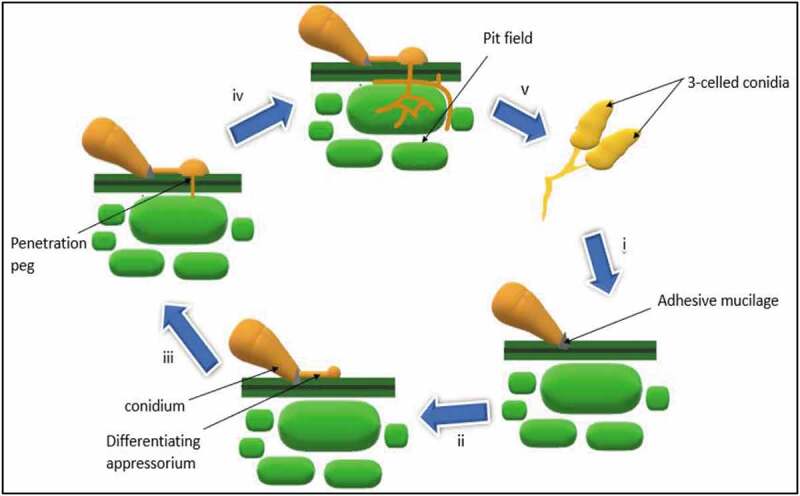
Infection cycle of *M. oryzae*: i) Attachment of conidia to the surface of host cell by adhesive mucilage ii) spore germination, development of germ tube and appressorium formation iii) emergence of penetration peg iv) invasive growth in the host plant. Modified from (Jia et al. 2016)

The *M. oryzae* infection cycle has distinct developmental stages (Figure 4). The first stage is characterised by the attachment of the three-celled conidium on the hydrophobic surface of rice leaf and development of polarised germ tube. In the second stage, this tube further divides into a swelled dome-shaped appressorium for the initiation of infection. Third stage embodies the development of the penetration peg from appressorium that is dependent on various ecological aspects, such as hydrophobicity and firmness of the interacting surface with negligible availability of exogenous nutrients. Further, the biotrophic infection spreads to the neighbouring cells, and infected cells enter a necrotrophic phase in which thin filamentous secondary hyphae eventually kill the plant cells (Ebbole 2007).

## Molecular targets in appressorium cell wall, signal transduction pathways and host-pathogen interactions

### Cell wall-associated factors

The pathogen *M. oryzae* penetrates into the plant cell wall through the melanin-rich dome-shaped appressorium. Melanin pigment plays an important role in imparting high turgor pressure to the appressorium, a major requirement for physical penetration and peg formation, a prerequisite to initiate infection (Galhano and Talbot 2011). Studies have shown that melanin-deficient fungal mutants with altered pigmentation (buffy, rosy, and albino) are non-pathogenic in nature (Langfelder et al. 2003). Polyketide synthase (PKS), encoded by the *alb1* gene, is the first enzyme of the melanin synthesis pathway. It converts acetate units into 1,3,6,8-tetrahydroxynaphthalene (4THN), the first stable intermediate. 4THN is reduced to scytalone by 1,3,6,8-tetrahydroxynaphthalene reductase (4HNR), then dehydrated to 1,3,8-trihydroxynapthalene (3THN) by the scytalone dehydratase (SD). The 3THN is further reduced to vermelone by 1,3,8-trihydroxynapthalene reductase (3HNR) and dehydrated by SD to yield 1,8-dihydroxynaphthalene (DHN) (Langfelder et al. 2003). Blocking the enzymes involved in the pathway could restrict the production of melanin, thus restricting the appressorium penetration into the leaf surface. The reductase enzymes, especially 3HNR, are known targets of tricyclazole (Thompson et al. 2000).

Glycerol accumulation is additionally required to generate appressorium turgor pressure that facilitates hyphal penetration to rupture plant cuticle. Glycerol synthesis requires mobilisation of lipid bodies (storage polymers) to the maturing appressorium (Galhano and Talbot 2011). The amount of glycogen is determined not only by its synthesis, but also by rate of its degradation. The cytosolic glycogen degradation in *M. oryzae*, which requires glycogen phosphorylase Gph1p, plays an important role in the virulence of the fungus (Badaruddin et al. 2013). Cleavage of the α-1,4-glycosidic linkages of glycogen can be achieved by phosphorolysis, catalysed by Gph1p, or by hydrolysis. The function of phosphorylase is to decrease glycogen levels during the early stationary phase. Earlier findings have shown that lack of *gph1* gene eliminated the transient drop in glycogen in the early stationary phase and resulted in an exaggerated re-synthesis phase (Badaruddin et al. 2013). The Δ*gph1* can prevent glycogen reserves from getting mobilised during development of appressorium and may significantly affect progression of disease.

## Signal transduction pathways

The primary metabolic variation during appressorium maturation are controlled by a *Tps* facilitated genetic switch, which response to glucose-6-phosphate levels and NADPH/NADP balance in the cell (Badaruddin et al. 2013). Earlier findings have suggested that mutants with altered multi-functional fatty acid β-oxidation protein *Mfp1* showed a considerable reduction in virulence (Wang et al. 2005). Moreover, mutants deficient in carnitine acetyltransferase enzyme meant for acetyl CoA transport across the mitochondrial or peroxisomal membrane, have been reported to be non-pathogenic (Wang et al. 2005).

**Figure 5.**
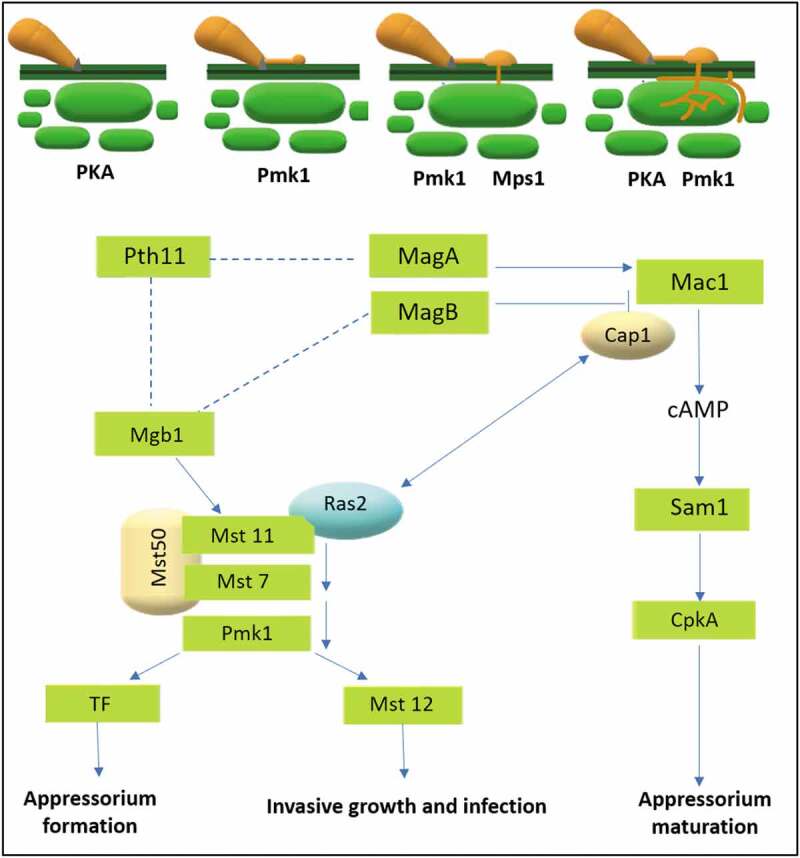
Schematic diagram of the Pmk1 mitogen-activated protein kinase (MAPK) pathway and the cyclic AMP (cAMP) response pathway in the rice blast fungus. Solid lines denote physical or genetic interactions that are supported by experimental evidence. Dotted lines denote tentative interactions Modified from (Wilson and Talbot 2009; Jiang et al. 2018)

The formation of appressorium on the artificial hydrophobic surfaces can be induced by exogenous cyclic Adenosine Monophosphate (cAMP). In absence of cAMP, conidia produce long germ tubes without tip differentiation on the hydrophilic surfaces. Molecular studies have confirmed the role of cAMP signalling in surface recognition and initiation of appressorium formation (Zhang et al. 2011). Besides surface hydrophobicity, other factors like surface hardness, cutin monomers and leaf waxes also affect appressorium formation in *M. oryzae* (Liu et al. 2007). Various physical and chemical signals have also been shown to affect appressorium formation in other plant pathogenic fungi, including *Ustilago maydis* and *Colletotrichum* species. It has also been reported that the *Cap1* gene is associated with the actin cytoskeleton involved in cAMP pathway, *Mac1* activation and transcription factors *Mst1, Som1, Cdtf1* function downstream from the cAMP–PKA pathway. Of these, *Som1* and *Cdtf1* are two novel transcription factors vital for sporulation and appressorium development (Liu et al. 2011). *Mst1* mutant has shown interrupted appressorium development due to delay in deployment of lipid bodies and transport of glycogens to appressoria, which is regulated by cAMP signalling (Soanes et al. 2012). There might be a cross-talk occurring with the cAMP pathway through the G-subunit protein *MagB*. The cAMP response pathway seems to be regulated by G proteins *MagA* and *MagB*, which potentially interacts with the *Pth11* G protein-coupled receptor. Adenylate cyclase, Mac1 causes the accumulation of cAMP, which binds to the regulatory protein kinase A subunit Sum1, allowing detachment of the catalytic subunit *CpkA* (Figure 5).

## Host-pathogen interaction

The rice-*M.oryzae* pathosystem has been extensively studied as a model for understanding plant-fungal interactions. Analysis of genetic and molecular interaction between avirulence effectors and their cognate resistance proteins have provided new insights into the resistance patterns in host plant as well as mode of pathogen infection. The formation as well as penetration of appressorium is regulated by *Pmk1* mitogen-activated protein (MAP) kinase (Zhang et al. 2011). The *Pmk1* MAP kinase gene plays a central role in appressorium formation and its growth beneath the plant cell surface. Its orthologs are also essential for numerous processes during plant infection stage in many other phytopathogenic fungi (Liu et al. 2011). A number of upstream genes involved in the activation of *Pmk1* MAP kinase have been identified, including *Mst50, Mst11, Mst7, Mgb1*, and *Ras2* genes (Zhao et al. 2005; Park et al. 2006).

*Mst50* functions as an adaptor protein that binds with both the *Mst7* and *Mst11* kinases. The *Mst7-Pmk1* interaction is a relatively transient weak interaction which may be stabilised or facilitated by additional components of the *Pmk1* MAP kinase pathway during appressorium formation (Ding et al. 2009)). The null mutant of *Pmk1* gene is defective in appressorium formation, infectious growth and has a lower phosphorylation level of *Pmk1*, but it still recognises hydrophobic surfaces and responds to exogenous cAMP (Figure 5). MAPK genes have also been reported to be essential for virulence, suggesting that components of MAPK signalling pathway involved in pathogenesis are widely conserved. The downstream transcription factor which regulates the *Pmk1* gene is *Mst12* involved in appressorial penetration and invasive growth (Li et al. 2011). Although key components of the cAMP signalling and Pmk1 pathways have been identified, fungal mechanisms for distinguishing physical and chemical signals of plant surfaces have not been well studied. The *Pth11* gene, one putative receptor gene has been reported to be involved in surface sensing in *M. oryzae* (Kramer et al. 2009; Rispail et al. 2009). The *Pth11* mutant demonstrated reduced virulence and appressorium formation on hydrophobic surfaces. The *M. oryzae* genome contains about 60 putative G-Protein Coupled Receptor (GPCR) genes, including several *Pth11*-like genes with the CEFM domain. Zhang et al. (2011) identified nine putative *Pmk1*-interacting genes, six and two of them being isolated from the appressorium and nitrogen starvation library, respectively. Only *Pic1* gene was identified in both the libraries. The authors predicted that some of these gene products may be involved in stimulating or stabilising the *Mst7-Pmk1* interaction during appressorium formation.

The firm attachment of spore and germ tube on the contact surface is one of the most important factors to induce appressorium differentiation. Another gene playing a significant role in surface recognition is *Cbp1*, which encodes a putative chitin-binding protein with signal peptide. The mutant study of *Cbp1* revealed formation of abnormal appressoria on artificial surfaces but normal functional appressorium formed on leaf surface (Kamakura et al. 2002; Liu et al. 2011). It has been observed that loss of the Cbp1p caused reduction in cellular attachment, suggesting that it might be a component of adhesive materials for germ tube attachment n (Kamakura et al. 2002; Kuroki et al. 2017).

The *Mpg1* gene of the fungal pathogen encodes a protein that modifies the leaf surface hydrophobicity before it adheres to it. Recombinant proteins under *in vitro* conditions can self-assemble on the surface of conidia (Xu et al. 2007), signifying that Mpg1p are capable of forming an amphipathic layer over surface of rice leaf, promoting adhesion of germ tube and acting as a signal for development of appressorium. Comparable to *Mpg1, Mhp1* is over expressed during plant colonisation and conidiation (Soonok et al. 2005). The *Mhp1* altered mutant had reduced conidiation, conidial propagation, appressorium development, and plant infection.

Therefore, various genes involved in the host–pathogen interaction can serve as key targets to stop the invasion and entry of pathogen in the rice plant if arrested at the point of initial contact.

## Targets of rice immunity

Various factors can influence plant immunity in response to the pathogen invasion. Plants possess an efficient immune system to protect them by detecting the conserved pathogen-associated molecular patterns (PAMPs) of invading pathogen and elicits PAMP-triggered immune response (Zipfel and Felix 2005) or through eliciting an effector-triggered immunity activated by cognate intracellular immune receptors (Jones and Dangl 2006). PAMP-triggered and effector-triggered immunity plays a crucial role in plant preinvasive and post-invasive resistance, respectively, and inhibits the colonisation of invading pathogen (Li et al. 2014; Lee et al. 2009b; Chen and Ronald 2011; Liu et al. 2012)

The rice plant possesses two types of resistance genes, responsible for blast resistance: a) Major resistance (R) genes that confer race-specific resistance and b) quantitative trait loci (QTLs) that control partial, nonrace-specific resistance (Skamnioti and Gurr 2009). The *Pik* locus having cluster of six alleles (*Pik, Pikm, Pikp, Piks, Pikh*, and *Pi1*) is of prime importance due to its role in a number of blast R genes involved in rice breeding (Zhai et al. 2011a). Recently, it has been demonstrated that polymorphic residues in *Pik-1* control the specificity of resistance (Carlos et al. 2018). Additional findings have recognised a new allele, *Pikx*, that is significantly associated with rice blast resistance at the *Pik* locus and is important for rice breeding against the *M. oryzae* (Shi et al. 2018). A total of 56 QTLs associated with blast resistance were identified in the rice genome (Hua et al. 2012; Liu et al. 2014). Only one QTL associated with resistance was present, and localised with the known R gene of *Pik* locus (Li et al. 2019).

Small interfering RNA (siRNA) and microRNA (miRNA) are expressed by plants under biotic stress conditions (Zhai et al. 2011b; Khraiwesh et al. 2012; Li et al. 2017a). The miRNAs present in rice have a vital role in providing immunity against associated pathogens. Innate immunity of rice is controlled by miR160a and miR398b against the blast fungus *M. oryzae* and their overexpression enhances resistance against blast disease (Li et al. 2014). Moreover, during the blast infection, dynamic balance of superoxide anion and hydrogen peroxide (H_2_O_2_) is maintained by superoxide dismutase2 targeted by miR398. MicroRNA miR169 acted as a negative regulator in rice immunity against *M. oryzae* by repressing the expression of nuclear factor *Y-A* genes (Li et al. 2017b). Likewise, jasmonic acid signalling pathway is a key regulator of plant defence responses against pathogens. *M. oryzae* infection blocks the conversion of α-linoleic acid to hydroxyoctadecadienoic acid, a critical step in jasmonic acid biosynthesis and facilitates its propagation and infection in the host plant. The miR319 is involved in the vital jasmonic acid biosynthetic step by genetic approaches (Zhang et al. 2018). OncemiR319 is induced in the host plant, specifically by the *M. oryzae* strain Guy11, which accomplishes the suppression of transcription factor gene *OsTCP21*, and its target genes *OsLOX2* and *OsLOX5* that encode key synthetic components of jasmonic acid (Zhang et al. 2018). Therefore, it has been suggested that miR319 may manipulate the plant innate response against *M. oryzae* by affecting jasmonic acid biosynthesis and signalling.

A recent study has also revealed an immune mechanism facilitated by phosphorylation of light-harvesting complex II (Liu et al. 2019). The light-harvesting complex II protein, LHCB5 in rice, has been reported to undergo light-induced phosphorylation at the time of blast infection. This step leads to accumulation of ROS in chloroplast, thereby enhancing broad-spectrum resistance of rice to *M. oryzae*.

## Eradication of rice blast from agricultural fields using Pesticides/Fungicides

The fungicides are applied as spray or powder to protect the plant foliage. These fungicides can be either contact/surface acting or systemic. The mode of their action can range from inhibition of protein synthesis to targeting cell membrane and respiration (Yang et al. 2011). There are a variety of chemicals used as pesticides/fungicides to suppress the infection of rice blast in the agricultural fields.

One of the earliest, efficacious agricultural antibiotics developed in Japan was Blasticidin-S (Takeuchi et al. 1958). Blasticidin-S has shown better activity against blast disease of rice as compared to other synthetic chemicals used. The antibiotic obstructs the protein synthesis in *M. oryzae* and acts at peptidyl transfer site on the ribosomes (Svidritskiy et al. 2013). However, application of Blasticidin-S by spraying is known to cause conjunctivitis by accidental interaction, therefore, an enhanced formulation consisting of calcium acetate is used to eliminate this risk. Blasticidin-S gets easily degraded in presence of sunlight and microorganisms in soil (Matsunaka et al. 2013), and no deposit is usually spotted in the grains of rice.

Kasugamycin, an aminoglycoside isolated from *Streptomyces kasugaensis* is used against bacterial grain rot of rice, seedling rot and rice blast caused by *Acidovorax avenae, Burkholderia glumae* and *M.oryzae*, respectively (Vakulenko and Mobashery 2003). Cytotoxicity of Kasugamycin is observed to be low with no phytotoxicity in majority of the harvests. Kasugamycin exerts its effect by binding to the 30S subunit of bacterial ribosome at the mRNA binding region, which in turn blocks the interaction between codon and anticodon at initiation of translation, thereby inhibiting tRNA binding and translation (Schluenzen et al. 2006; Schuwirth et al. 2006).

Sodium hypochlorite has also been used to eliminate blast infection on seeds. Seeds soaked in low concentrations (0.8%-3.2%) of sodium hypochlorite were successful in reducing blast infection. Moreover, sodium hypochlorite concentrations and soak time required to eliminate *P. grisea* blast from infested seed resulted in stunted and chlorotic seedlings. The germination rates were reduced from >90% to <75% and seedling health was visibly reduced (Greer and Webster 2001).

Azoxystrobin is the most commonly used rice fungicide in the southern United States and Asian countries (Groth 2005). Information is limited on the optimum rates and timing of fungicide application for blast control as compared with sheath blight information (Prasanna et al. 2013). Timing of fungicide application is critical and is targeted towards protecting the panicle as it emerges from the flag leaf. Consequently, there has been a general trend to make a single application of azoxystrobin in attempt to control both blast and sheath blight (Groth 2005). It is an effective inhibitor of spore germination and is most effective when used as a protectant prior to infection (Bartett et al. 2001). Preliminary studies indicate the rate of azoxystrobin required to effectively control blast infections, as disease incidence varies with the susceptibility of the host (Uppala and Zhou 2018).

## Pesticides targeting biosynthesis of phospholipids in rice blast

Iprobenphos (IBP) has been popularly used for controlling blast disease of rice along with Edifenphos (EDDP) after phosphorothiolate (PTL) compounds were discovered to exhibit fungicidal properties. IBP is a systemic fungicide, and EDDP is non-systemic fungicide showing effective fungicidal activity when used as a foliar applicant against the blast causing fungus (Gohel and Chauhan 2015). Isoprothiolane, malonate ester having two methylene hydrogens is also used as a fungicide to control a range of diseases including blast of rice. The chemical structure of PTL is apparently different from that of isoprothiolane, yet, cross-resistance between PTL fungicides and isoprothiolane proposes a parallelism in their mode of action (Fukuta et al. 2004). IBP, EDDP and isoprothiolane have shown to explicitly prevent the conversion of phosphatidylethanolamine to phosphatidylcholine (Upmanyu and Rana 2012). Inhibition of phosphatidylcholine synthesis leads to increased permeability in the cell membrane along with enzymatic activities that are membrane-associated, detrimental to fungal pathogen. Additionally, propiconazole inhibits sterol biosynthesis by inhibiting demethylation of ergosterol important for cellular growth of the fungus (Kumar and Veerabhadraswamy 2014; Uppala and Zhou 2018).

## Pesticides for disrupting respiration and permeability of the membrane

Novel systemic fungicides, ferimzone and metominostrobin are also used to inhibit blast of rice. Fungistatic action of ferimzone *in vitro* involves efflux of acidic electrolytes from the mycelia of *M.oryzae* (Kawasaki 2004). Metominostrobin, belonging to the class of methoxyacrylate fungicides, prevents the electron chain during mitochondrial respiration by obstructing the flow of electron via the cytochrome *bc1* segment. However, mycelial cells recover respiratory activity by inducing cyanide-resistant respiration to release the block by metominostrobin. Metominostrobin-dependent initiation mechanism is proposed to be dependent on the superoxide anion during cyanide-resistant respiration. When applied, the flavonoids have also been able to scavenge superoxide anion thus generated, by blocking the flow of electron through the cytochrome *bc1* segment, thereby inhibiting metominostrobin-dependent induction of cyanide-resistant respiration (Yamaguchi 2004). Similarly, another chemical Kresoxim methyl, contact or local in nature, disrupts the respiration by blocking the electron transport by binding to the Q_o_ site of the chain (Kumar and Veerabhadraswamy 2014).

Eprobenfos is another systemic fungicide that alters the structure of the membrane by blocking the phospholipid synthesis, thereby increasing the permeability and causing loss of key cellular components (Srivastava et al. 2017).

## Melanin biosynthesis inhibition

**Figure 6.**
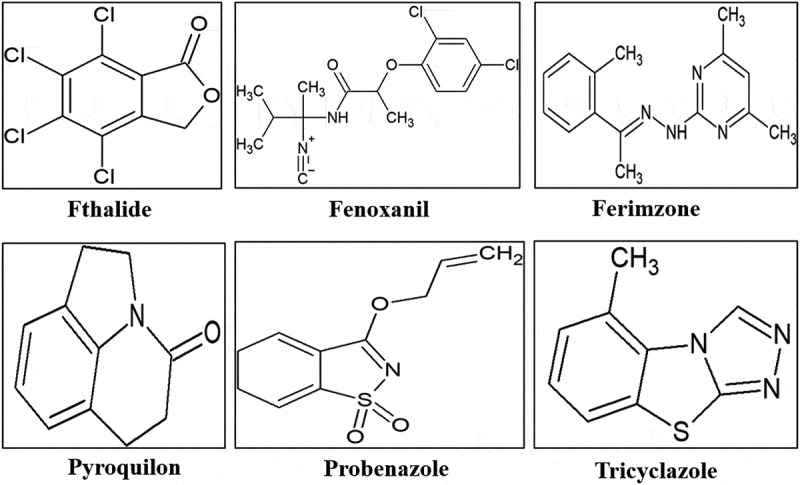
Structural representation of various pesticides used as inhibitors against rice blast infections

Appressoria cells formed during the infection cycle of *M.oryzae* are matured by the development of a layer of melanin, which facilitates the generation of necessary turgor pressure required for its penetration into leaf. Thus, inhibitors of melanin biosynthesis have been reported to show a remarkable effect as blast controller (Kimura and Fukuchi 2018). Hydroxynaphthalene inhibitors and scytalone dehydratase inhibitors are the two categories of melanin biosynthesis inhibitors. The structure of inhibitors is shown in (Figure 6). Fthalide has an effective defensive action against rice blast for long duration (Yamaguchi 2004). It restricts melanin biosynthesis in appressoria crucial during invasion process of *M. oryzae*. Phytotoxicity as well as cytotoxicity to mammals from the main metabolites has been indicated to be negligible.

Tricyclazole, is a reductase inhibitor which results in the deposition of auto-oxidation intermediate products of the pathway (Kunova et al. 2013). It inhibits melanin biosynthesis not only in *M. oryzae* but also in *C. lindemuthianum* and *C. lagenarium* (Sisler 1986). Pyroquilon exerts similar modes of action *i.e*. scytalone and 2-hydroxyjuglone (2-HJ) accumulation in the cell. Increased concentrations of tricyclazole followed by pyroquilon induce flaviolin accumulation, signifying that alternative step of inhibition is present in the pathway. The chemical also obstructs secondary infection by decreasing the sporulation of *M. oryzae* under field settings. Field studies indicated long-lasting inhibitory potential of tricyclazole and pyroquilon (Kunova et al. 2014).

Carpropamid has been established as an effective regulatory root systemic agent against the rice blast. Various studies revealed that it is a competitive inhibitor that has tight-binding with an enzyme in the melanin biosynthesis pathway, scytalone dehydratase (STD). Other systemic fungicides that function as inhibitors to melanin biosynthesis are pyroquilone, diclocymet and fenoxanil (Nishimura and Hino 2002; Srivastava et al. 2017). Tebuconazole also acts as inhibitor of fungal cell wall development in addition to melanin inhibition (Ghazanfar et al. 2009).

## New approaches to combat rice blast disease

### Mutation breeding

The mutated genes with the help of tagging can be bought into a noble solitary breeding line making it easier to track consequent breeding programme (Shu 2009). The isolation and molecular characterisation of different blast resistance genes can lead to clarification of the actual allelic variants of these genes via various molecular breeding and transgenic approaches. The bioinformatics approach is helpful in understanding the evolution of new pathotypes of *Magnaporthe* isolates by studying major blast resistance (R) gene. This approach is utilised for designing better resistance breeding strategies. In this context, allele mining for resistance genes in all sequenced rice genomes shows the presence/absence of polymorphism and a large number of structural variations (Gowda et al. 2015; Mahesh et al. 2016). Approximately 100 quantitative blast R genes have been detected in rice, and 22 of these have been successfully cloned and characterised (Sharma et al. 2012; Ashkani et al. 2016). There are a number of varieties which have been formed by mutation breeding, like RD6, glutinous mutant of the prominent non-glutinous variety Khao Dawk Mali 105 (KDML105) induced by radiation. Chemo- mutagenesis with 0.1 and 0.2% ethylmethane sulphonate (EMS) has also been deployed to impart blast resistance in the Ratna (IR8/TKm6) variety (Kumar et al. 2017). However, the drawback of mutation breeding while protecting crop against rice blast infection is restricted in effecting the production of dominant alleles with less efficacy.

## Resistance to blast through Marker-assisted selection (MAS)

MAS in comparison to the traditional breeding approaches, is convenient in breeding for acquiring resistance to blast, as only single or few genes are involved in coding of these resistant phenotypes (Srivastava et al. 2017). It is a suitable approach to control blast by manipulating the interaction among specific R gene and *Avr* gene of avirulence from host–pathogen interaction (Petit-Houdenot and Fudal 2017; Srivastava et al. 2017). MAS along with conventional breeding has facilitated R genes to be combined in elite rice varieties to improve their blast resistance and durability.

MAS also improves the success rate of traditional breeding by selecting those that could assist in achieving the required traits. A group of simple sequence repeats markers viz. RM168, RM8225, RM1233, RM6836, RM5961 and RM413, that have been reported to be linked to blast immunity trait, can be further utilised in MAS programs (Ashkani et al. 2012). Limitations of MAS programme are the high costs involved and less consistency, since they hamper the effective application for varietal development. Mapping and tagging of QTLs associated with blast resistance would be supportive in the cloning of major disease resistance genes as well as marker-assisted breeding program for development of resistant cultivars (Ashkani et al. 2012).

## Blast disease management by miRNA

MicroRNAs (miRNAs) are one of the vital regulators for development and defence in eukaryotic species. miRNAs are implicated in providing immunity against spread of pathogenic organisms in plants, which could be used as biological control agents (Katiyar-Agarwal and Jin 2010). Expression of nine miRNAs has been identified upon infection by *M. oryzae* after inoculating with *M. oryzae* elicitors (Li et al. 2014). The involvement of miRNAs in providing resistance to rice plant against *M. oryzae* has been reported by (Katiyar-Agarwal and Jin 2010. Li et al. 2017b) also elucidated that the increased expression of *miR398b* or *miR160a* can improve immunity against the rice blast disease (Li et al. 2017b). Systematic silencing of *M. oryzae* has been directed and used for the improvement of rice plant resistance. The *OsACDR1* gene is a resistant gene that encodes for MAPKKK, involved in producing defence-related pathways aimed at up-regulating the *OsACDR1* transcript (Srivastava et al. 2017). Rice plants with increased expression of *OsACDR1* illustrated impulsive hypersensitive response and heightened build-up of the phenolic compounds, required for the positive alteration leading to gain of resistance in the plant (Srivastava et al. 2017).

## Transgenic approaches for blast resistance

Effective and stable integration of gene of interest into the rice genome has been confirmed through *Agrobacterium-*mediated transformation (Srivastava et al. 2017). Studies have shown aggravated resistance in rice cultivars against blast fungus by expressing chitinase gene in rice by the use of these approaches (Li et al. 2009). Insertion of *RCC2* by the above-mentioned approach has shown that resistance levels increased in rice under *in-vitro* conditions (Asghar et al. 2007). Increasing the level of gene expression in transformed rice for *OsCPK4* encoding for calcium-dependent protein kinase in transgenic rice gave resistance against the blast infection. Numerous significant genes have been effectively mutated in the selected varieties of rice for managing the blast disease in rice (Srivastava et al. 2017).

## Perspective

Rice blast, caused by *M. oryzae*, is one of the most destructive rice diseases worldwide, causing substantial yield losses every year. Traditional strategies to manage this disease include the fertilisation of the different cultivars properly; usage of high quality and disease-free seeds etc. Besides, the other techniques used to manage blast infections are seed treatments (to prevent infection of the seedlings after germination) and application of fungicides in the fields (to prevent infection of leaves and panicles during the growing season). These techniques have been used to manage and reduce rice blast, but neither one is considered to be highly successful. Recent advances in molecular techniques have been used as an effective tool to understand the biological pathways as well as genes taking part in host-pathogen infection pattern, plant response and disease development.

The understanding of major molecular targets associated with cell wall, signal transduction pathways and host–pathogen interactions in *M. oryzae* could help to prevent the onset of blast disease, limiting the use of harmful chemicals as pesticides. Recent practices like mutation breeding, MAS for blast resistance in rice programme, miRNA in blast disease management, transgenic approaches for blast resistance can also play key role in monitoring blast disease.
